# Supplementary material for: Splice-Junction-Based Mapping of Alternative Isoforms in the Human Proteome
Source: Cell Rep. Author manuscript; Available in PMC 2020 Jan 15. (PMC6961840; doi:10.1016/j.celrep.2019.11.026)
Supplement: 2 [file NIHMS1546469-supplement-2.zip › DF1/PXD006675/RightVentricle/RightVentricle_19_TTN_SNCTVSVHVSEPPIFR.pdf]

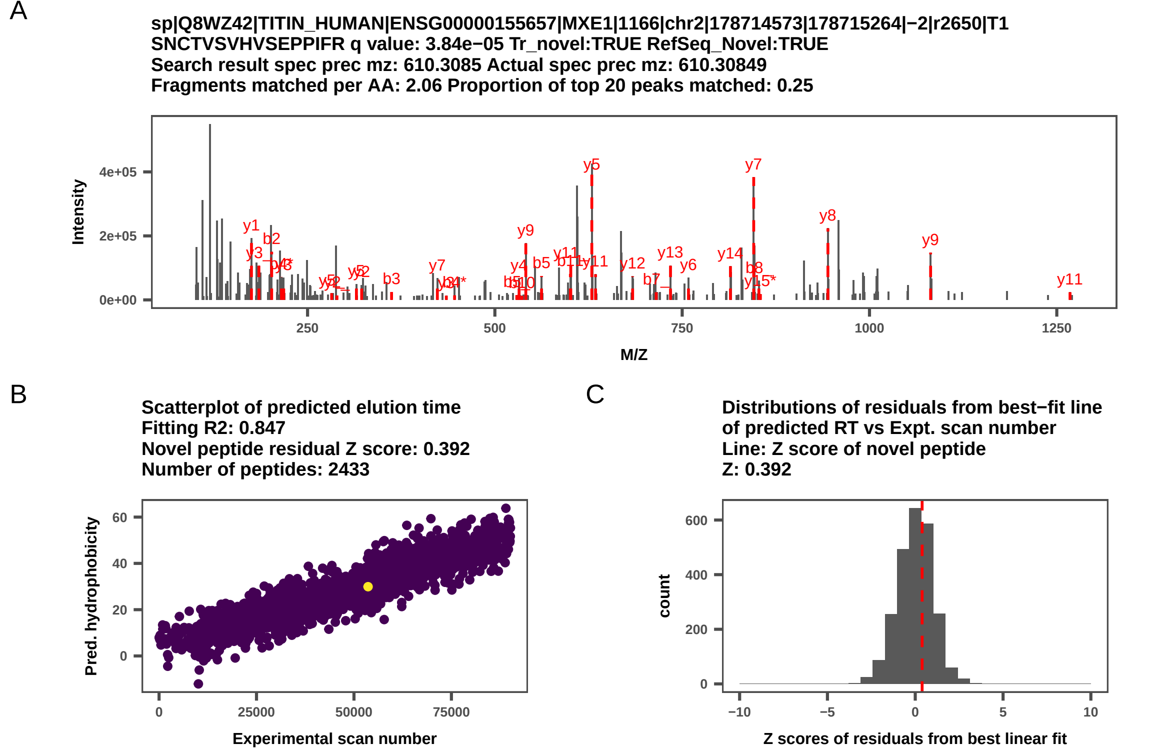
A
sp|Q8WZ42|TITIN_HUMAN|ENSG00000155657|MXE1|1166|chr2|178714573|178715264|−2|r2650|T1
SNCTVSVHVSEPPIFR q value: 3.84e−05 Tr_novel:TRUE RefSeq_Novel:TRUE
Search result spec prec mz: 610.3085 Actual spec prec mz: 610.30849
Fragments matched per AA: 2.06 Proportion of top 20 peaks matched: 0.25
Intensity
4e+05
2e+05
0e+00
y1
b2
y3
y5
y7
y8
y9
y11
y12
y13
y14
y6
b3
b5
b7
b8
y4
M/Z
250
500
750
1000
1250
B
Scatterplot of predicted elution time
Fitting R2: 0.847
Novel peptide residual Z score: 0.392
Number of peptides: 2433
Pred. hydrophobicity
60
40
20
0
0
25000
50000
75000
Experimental scan number
C
Distributions of residuals from best−fit line
of predicted RT vs Expt. scan number
Line: Z score of novel peptide
Z: 0.392
count
600
400
200
0
−10
−5
0
5
10
Z scores of residuals from best linear fit
